# Psychogenic Polydipsia: The Result, or Cause of, Deteriorating Psychotic Symptoms? A Case Report of the Consequences of Water Intoxication

**DOI:** 10.1155/2015/846459

**Published:** 2015-01-21

**Authors:** Melissa Gill, MacDara McCauley

**Affiliations:** ^1^Cavan/Monaghan Mental Health Services, Drumalee Primary Care Building, Cootehill Road, County Cavan, Ireland; ^2^St. Brigid's Hospital, Ardee, County Louth, Ireland

## Abstract

Water intoxication is a rare condition characterised by overconsumption of water. It can occur in athletes engaging in endurance sports, users of MDMA (ecstasy), and patients receiving total parenteral nutrition. This case outlines water intoxication in a patient with psychogenic polydipsia. When the kidney's capacity to compensate for exaggerated water intake is exceeded, hypotonic hyperhydration results. Consequences can involve headaches, behavioural changes, muscular weakness, twitching, vomiting, confusion, irritability, drowsiness, and seizures. Cerebral oedema can lead to brain damage and eventual death. In this case, psychogenic polydipsia led to significant hyponatraemia, cerebral oedema, and tonic-clonic seizures. Differential diagnoses for hyponatraemia are outlined. The aetiology of psychogenic polydipsia is uncertain, but postulated hypotheses are explored. Psychogenic polydipsia occurs in up 20% of psychiatric patients and this case serves to remind us to be cognizant of water overconsumption.

## 1. Introduction

Water intoxication is a rare condition characterised by overconsumption of water. In the case outlined below, it emerged as a result of psychogenic polydipsia. Other scenarios can include athletes engaging in endurance sports, users of MDMA (ecstasy), and iatrogenic causes, for example, patients receiving total parenteral nutrition. This case describes a patient who, as result of psychogenic polydipsia, developed serious consequences of hyponatraemia. With psychogenic polydipsia occurring in up to 20% of psychiatric patients, the potentially fatal consequences cannot be ignored. Although psychogenic polydipsia has been previously described in the literature, this case highlights the fact that a common symptom can be trivialized if not regularly highlighted. We believe that this case report serves as a reminder of an important clinical lesson.

## 2. Case Presentation

A 43-year-old man with a previous diagnosis of bipolar disorder and alcohol dependence was admitted as an involuntary patient. He had been agitated and was irritable prior to admission, engaging in uncharacteristic behaviors such as blowing smoke in his son's face and kicking the family pet. He admitted to feeling paranoid that people were talking about him and described poor sleep, appetite, energy, and concentration. He appeared perplexed and thought-disordered and displayed poor insight, repeatedly expressing a desire to be discharged home. Medications on admission included nortriptyline 30 mg nocte and zopiclone 7.5 mg nocte. Risperidone 3 mg nocte was commenced.

He had had six admissions to hospital in the three years since his diagnosis and had completed twelve sessions of ECT for a prolonged depressive episode just three months prior to this admission. During the course of his admission, his behaviour deteriorated significantly. He refused to attend to self-care, exposed his genitals on a number of occasions, and attempted to masturbate in front of patients and staff. Because of his unmanageable behaviour, he was briefly secluded on two occasions. Risperidone was changed to olanzapine, nortriptyline was discontinued, and sodium valproate was commenced.

It was noted by staff that the patient was consuming copious quantities of water on the ward. He was also witnessed to self-induce vomiting and, on questioning, stated that he was drinking excessively in order to induce vomiting. He also expressed concerns about frequent micturition. Sodium levels were checked and were found to be 128 mmol/L on day 40 of admission. In light of his escalating abnormal behaviour, a CT scan was requested and this was performed on day 53 of admission. An initial verbal report indicated that no abnormalities were detected. On the same day, his sodium valproate was reduced and subsequently stopped on day 56, due to lack of response. Clinical notes indicated that the patient's behaviour continued to deteriorate. He became increasingly disinhibited in the days after his CT scan, exposing himself to staff and fellow patients. The deterioration in his behaviour culminated in his urinating publicly on the ward and at the statutory review of his involuntary detention, he reported that God had instructed him to do so.

On day 57 of admission, the patient was witnessed by nursing staff having a tonic-clonic seizure. The doctor on duty was present on the ward and an ambulance was immediately called. The seizure was prolonged, failing to respond to two administrations of rectal Diazepam prior to the arrival of the ambulance. The patient was transported to the local general hospital where he underwent a second CT scan, showing significant cerebral oedema this time. On admission his level of sodium was 108 mmol/L. He was treated in ICU for 6 days and was discharged home following psychiatric assessment in the general hospital. His psychiatric care was taken over by the local Home Based Treatment Team. He has remained seizure-free and is being successfully treated in the community from a mental health point of view. He was not offered general medical follow-up.

## 3. Discussion

Hyponatraemia is defined as a plasma sodium level below 135 mmol/L, but symptoms such as lethargy, restlessness, and disorientation generally only occur once plasma levels drop to 115–120 mmol/L. Symptoms of hyponatraemia rarely occur unless patients continue to drink excessive amounts of water (>10 litres/day) after maximum urine dilution is reached (100 mOsm/kg with minimum urine osmolality) and the antidiuretic hormone of the patients is fully suppressed [1]. When the kidney's capacity to compensate for exaggerated water intake is exceeded, hypotonic hyperhydration results. The consequences can involve headaches, behavioural changes, muscular weakness, twitching, vomiting, confusion, irritability, drowsiness, and seizures. Cerebral oedema can lead to brain damage and eventual death [2]. As in the case outlined above, tonic-clonic seizures are the most commonly identified presenting symptom, occurring in up to 80% of initial presentations [3].

Differential diagnoses for hyponatraemia include SIADH, diabetes insipidus, hyperthyroidism, and excess cortisol. Extreme water consumption, as in the case outlined, is also implicated [4]. Psychogenic polydipsia is a disorder that can lead to significant morbidity and mortality and occurs in 6% to 20% of psychiatric patients [1, 5]. Although psychogenic polydipsia is relatively common in this population, only one-fifth to one-third of polydipsic patients will experience symptomatic hyponatraemia [6]. A number of psychiatric disorders have been linked with psychogenic polydipsia. The most commonly reported psychiatric disorder is chronic schizophrenia, but it may also occur in anorexia nervosa [2] and psychotic depression and bipolar psychosis [7, 8]. A specific link to previous alcohol misuse has been found [9], a diagnosis which, along with bipolar disorder with psychotic symptoms, applies to the patient described in this report. The latter study found rates of alcohol abuse over three times greater in schizophrenic patients with polydipsia than in those without.

The aetiology of psychogenic polydipsia is uncertain and is likely multifactorial [1]. Impaired water excretion and water intoxication were noted in the psychiatric literature of the early 20th century, prior to the introduction of antipsychotic medication. The antidiuretic hormone, arginine vasopressin (AVP), has been implicated. In normal circumstances, the brain carefully regulates the concentration of solute in tissues, maintaining it within a very narrow range by controlling the secretion of AVP from the hypothalamus. Irrespective of fluid intake, this system can precisely regulate the internal milieu. Evidence suggests that the osmotic set point for AVP secretion may be lower in patients with polydipsia and hyponatraemia, leading to impairment in water excretion. Psychological stress and acute psychosis may contribute to this transient resetting of the osmostat [10]. Other postulated hypotheses include stimulation of thirst centres by elevated dopamine levels, drinking to counteract anticholinergic side effects of psychotropic medications, and changes in feedback regulation of the hypothalamic-pituitary axis induced by chronic polydipsia [1].

Antipsychotics have been recognised to lower the seizure threshold [11], including olanzapine, which was prescribed to the patient in this study [12]. This may have played a part in the above case. The patient had had ECT three months prior to the admission, but a literature search failed to indicate any link between ECT and hyponatraemia. No EEG had been carried out at baseline. Sodium valproate, with its antiepileptic properties, had been stopped the day before the seizure occurred. Also interesting is the observation that hyponatraemia can worsen psychotic symptoms and early signs of sodium deficiency may mimic psychosis [13] or bipolar disorder [14]. This has been more commonly recognised in the elderly but may apply to the patient described, who was sufficiently well once his hyponatraemia was corrected to be treated, albeit intensively, in the community.

This case highlights the potentially catastrophic effects of psychogenic polydipsia. The phenomenon is not a new one, but this case highlights a slightly different viewpoint which is that whether hyponatraemia is an independent result of psychogenic polydipsia, or whether indeed it, in itself, introduces a vicious circle, leading to hyponatraemia that causes many of the psychiatric sequelae. In the case described, the patient's behaviour significantly deteriorated between the date of his normal CT scan and the day of his seizure. The deterioration was attributed purely to his psychosis, but we argue that consideration must be given to the possibility, in such patients, that the symptoms are generated by a physical cause, hyponatraemia in this case, rather than assuming that the original psychiatric illness is to blame.

Given how common psychogenic polydipsia is, we suggest that routine enquiry should be made into excessive water intake, in both inpatient and outpatient settings. Nursing staff, who observe patients continuously on a ward, should be educated about the importance of detection and management of the condition. Bear in mind that the differential diagnosis of symptoms of polyuria and polydipsia includes diabetes insipidus and mellitus, Addisonian crisis, Conn's syndrome, and chronic renal failure [2]. In general, sodium levels between 130 and 135 mmol/L are asymptomatic, and decisions regarding referral or assessment by a medical team should be based on the patient's overall condition. Hospital admission is generally necessary for many patients with sodium levels below 130 mmol/L and for almost all with sodium less than 125 mmol/L. Neurological signs in conjunction with hyponatraemia always necessitate referral [15]. Caution must be taken in correction of hyponatraemia as central pontine myelinolysis may occur if correction is overly rapid. Guidelines suggest a correction rate of less than 10 mmol/L over the course of 24 hours [16]. In terms of management of persistent psychogenic polydipsia, therapeutic options such as strict fluid restriction and behavioural approaches have been found to be effective in reducing its severity [17].
